# Supplementary material for: Metabolic priming by multiple enzyme systems supports glycolysis, HIF1α stabilisation, and human cancer cell survival in early hypoxia
Source: EMBO J. 2024 Mar 14;43(8):1545–69. doi: 10.1038/s44318-024-00065-w (PMC11021510; doi:10.1038/s44318-024-00065-w)
Supplement: Supplementary file 1 — Appendix [file 44318_2024_65_MOESM1_ESM.pdf]

APPENDIX

Grimm *et al.*

Table of contents

Appendix Figures

Appendix Figure S1, related to Figure 1……………………………………………………..………………..2
Appendix Figure S2, related to Figure 2……………………………………………………..………………..3
Appendix Figure S3, related to Figure 3……………………………………………………..………………..4
Appendix Figure S4, related to Figure 4……………………………………………………..………………..6
Appendix Figure S5, related to Figure 5……………………………………………………..………………..8
Appendix Figure S6, related to Figure 6……………………………………….……………..……...……..10

Appendix Tables

Appendix Table S1. Table of fragment ions used for metabolite quantification by GC-MS…………...12
Appendix Table S2. Table of fragment ions used for metabolite quantification by LC-MS……………13

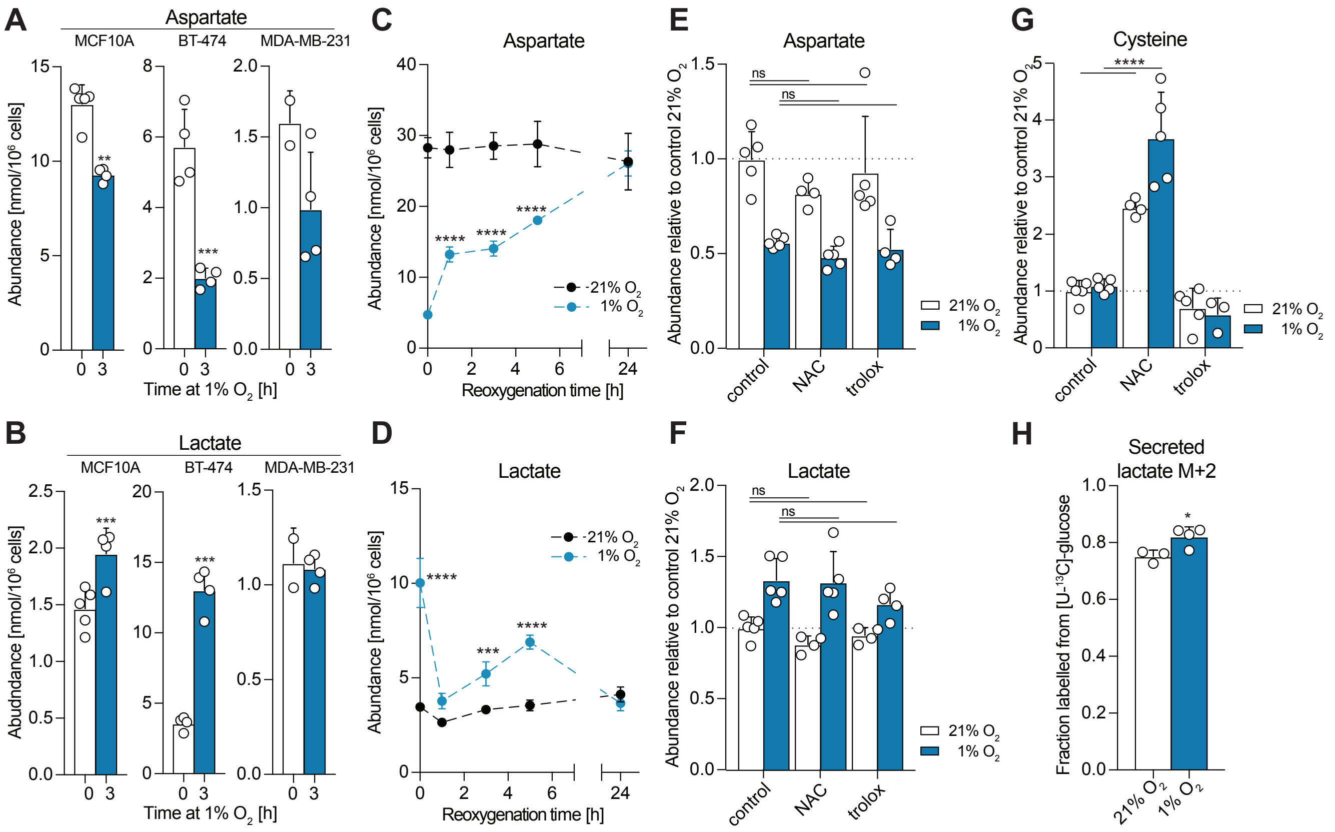

Appendix Figure S1 related to Figure 1. Increase in lactate and decrease in aspartate levels induced by exposure to 1% O_2 occur in other cell lines, are reversible upon reoxygenation and cannot be prevented by antioxidant treatment.

A-B) Intracellular abundances of lactate and aspartate in the indicated cell lines at 21% O_2 and after 3 h at 1% O_2 See also **Figure 1C-D**.

C-D) Intracellular abundances of lactate and aspartate in MCF7 cells exposed to 1% O_2 for 3 h, followed by reoxygenation at 21% O_2 for the indicated lengths of time, compared to control cells in normoxia.

E-G) Intracellular abundances of lactate, aspartate and cysteine in MCF7 cells incubated in 21% O_2 or 1% O_2 for 3 h, with and without antioxidant treatment (NAC, N-acetylcysteine: 1mM; Trolox: 50 µM. Cysteine levels shown to confirm the effect of NAC on cells.

H) Fraction of lactate M+2 labelled from [U-^{13}C]-glucose in cell culture media from MCF7 cells incubated in 21% O_2 or 1% O_2 for 5 h with the isotopic tracer. The quantified ion (m/z 117) contains two carbons that correspond to C2 and C3 of lactate.

Data information: Datapoints in all panels represent mean ± s.d. n = 5 (E, G), n = 3 (H) or n = 4 (A-D) cultures per cell line/time point/condition [except in A, B; MDA-MB-231 0 h (n = 2) and MCF10A 0 h (n = 5)]. P values for differences between 21% vs 1% O_2 were calculated by a two-tailed unpaired t-test (A, B, H), two-way ANOVA Sidak's test (C-D) or two-way ANOVA Dunnett's test (E, G). ns: non-significant, * P<0.05, ** P<0.01, *** P<0.001, **** P<0.0001.

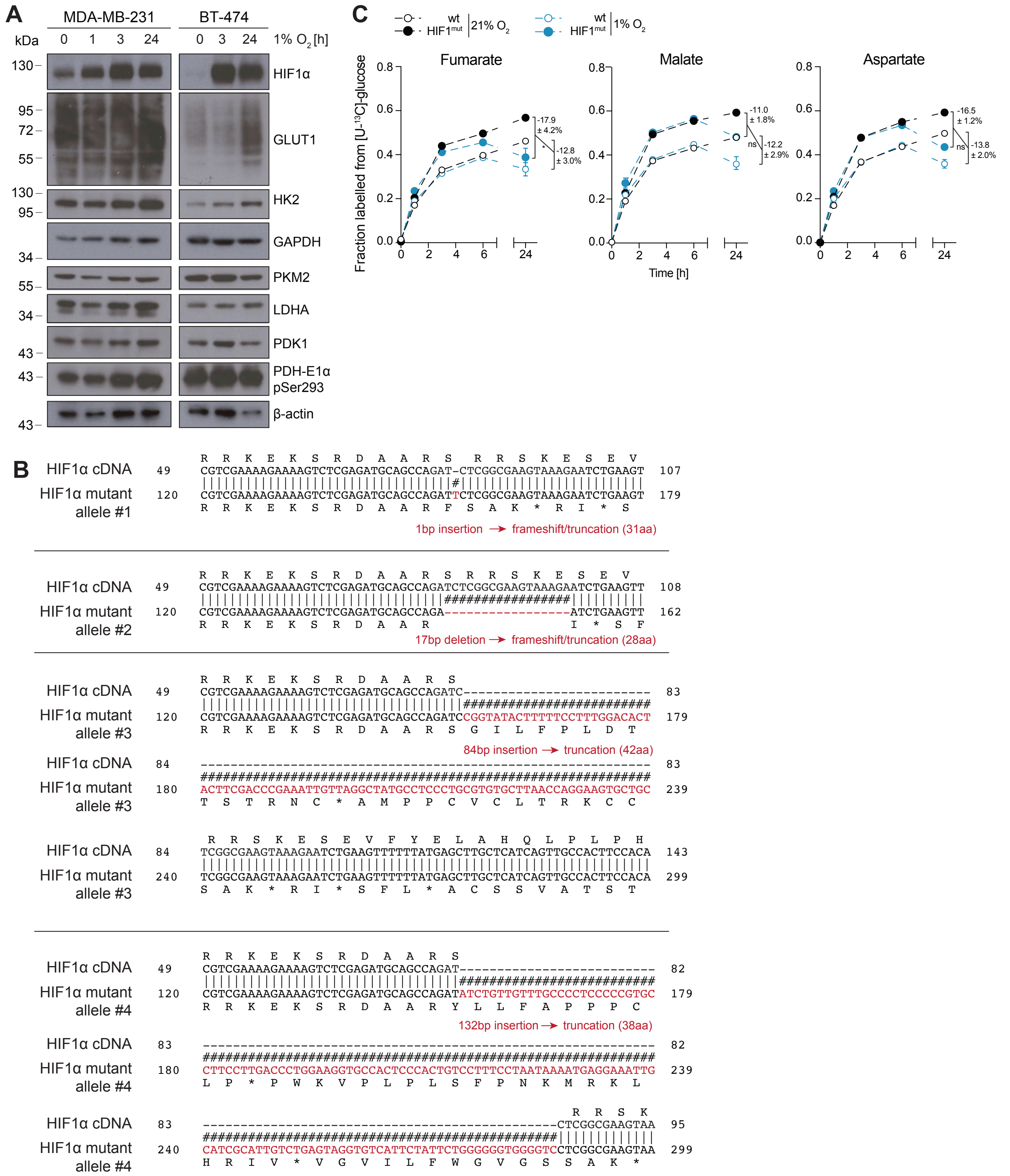

Appendix Figure S2 related to Figure 2. HIF1α target expression in breast cancer cell lines and metabolic response to hypoxia in HIF1α^{mut} MCF7 cells.

A) Western blot to assess levels of HIF1α and a panel of HIF1α targets in the indicated cell lines incubated in 21% O_2 or at 1% O_2 for the indicated lengths of time. See also **Figure 2A**.

B) Sequence alignment of mutant HIF1α alleles in HIF1α^{mut} MCF7 cells with exon 2 of human HIF1α cDNA (CCDS9753.1).

C) Fraction of the indicated metabolite pools labelled from [U-^{13}C]-glucose in wild-type (wt) and HIF1α^{mut} MCF7 cells as in **Figure 2D**. See also **Figure 2D**.

Data information: Datapoints in C represent mean ± s.d. and statistical errors were propagated to calculate variance of the change in isotopic labelling between normoxia and hypoxia for each cell line. n = 4 cultures for each time point and condition. The P values shown were calculated by two-way ANOVA Sidak's test. ns: non-significant, * P<0.05, ** P<0.01, *** P<0.001, **** P<0.0001.

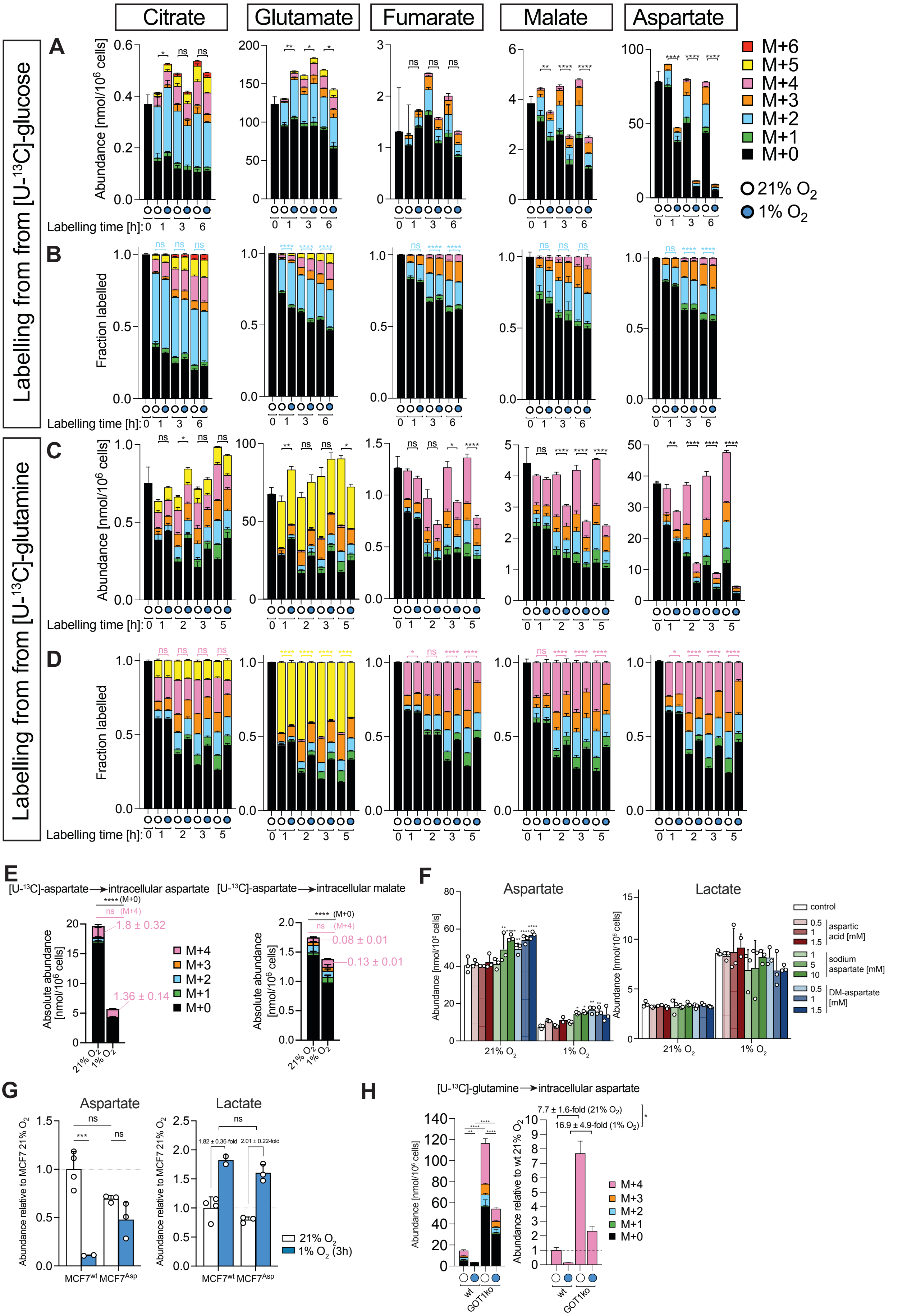

Appendix Figure 3 *- Figure legend on the next page*

Appendix Figure S3 related to Figure 3. Evidence for decreased aspartate synthesis from both glucose and glutamine, and fate of exogenous aspartate in early hypoxia.

A-B) Absolute (A) and fractional (B) isotopologue abundances of the indicated metabolites after incubation of MCF7 cells with [U-^{13}C]-glucose in 21% O_2 or 1% O_2 for the lengths of time shown. Data are from the same experiment as in **Figure 2D** and **S2C**, shown here for comparison with glutamine labeling in panel **C** below.

C-D) As in **A** and **B**, respectively, but using [U-^{13}C]-glutamine.

E) Absolute isotopologue abundance distribution of intracellular aspartate (left) and malate (right) in MCF7 cells incubated with 1.5mM [U-^{13}C]-aspartate.

F) Intracellular abundances of aspartate and lactate in MCF7 cells in 21% O_2 and after 3 h in 1% O_2. Media were supplemented with the indicated concentrations of aspartic acid, sodium aspartate or cell-permeable dimethyl-aspartate (DM-aspartate) for 3 h.

G) Intracellular aspartate and lactate abundances in MCF7 cells, or cells that had been chronically cultured in 0.5mM aspartate (MCF7^{Asp}), incubated in 21% O_2 or 1% O_2 for 3 h in the presence of 0.5mM aspartate.

H) Aspartate isotopologue abundance distribution in wt or GOT1ko MCF7 cells incubated with [U-^{13}C]-glutamine in 21% O_2 or 1% O_2 for 3h. The bar graph on the right shows only the M+4 isotopologue abundance relative to wt cells in 21% O_2.

Data information: Datapoints in all graphs represent mean ± s.d. (incl. error propagation in H). n = 4 [A-D (except 1% O_2 6h (n = 3), H], n = 5 (E), n = 2-3 (F) cultures for each time point and condition. For panel G, n = 4, 2, 3, 3 (for MCF7^{wt} 21% O_2, MCF7^{wt} 1% O_2, MCF7^{Asp} 21% O_2 and MCF7^{Asp} 1% O_2, respectively) cultures per time point and condition. P values shown were calculated by two-way ANOVA Sidak's test (A, C, E, G-aspartate), two-way ANOVA Tukey's test (B, D, H-left), two-way ANOVA Dunnett's test (F), or a two-tailed, unpaired t test (G-lactate, and H-right for significance of fold-change differences). For A-D, only the P values for the major isotopologues generated by each label are shown (for B: M+2 of all metabolites and for D: glutamate M+5; and citrate, fumarate, malate, aspartate M+4. ns: non-significant, * P<0.05, ** P<0.01, *** P<0.001, **** P<0.0001.

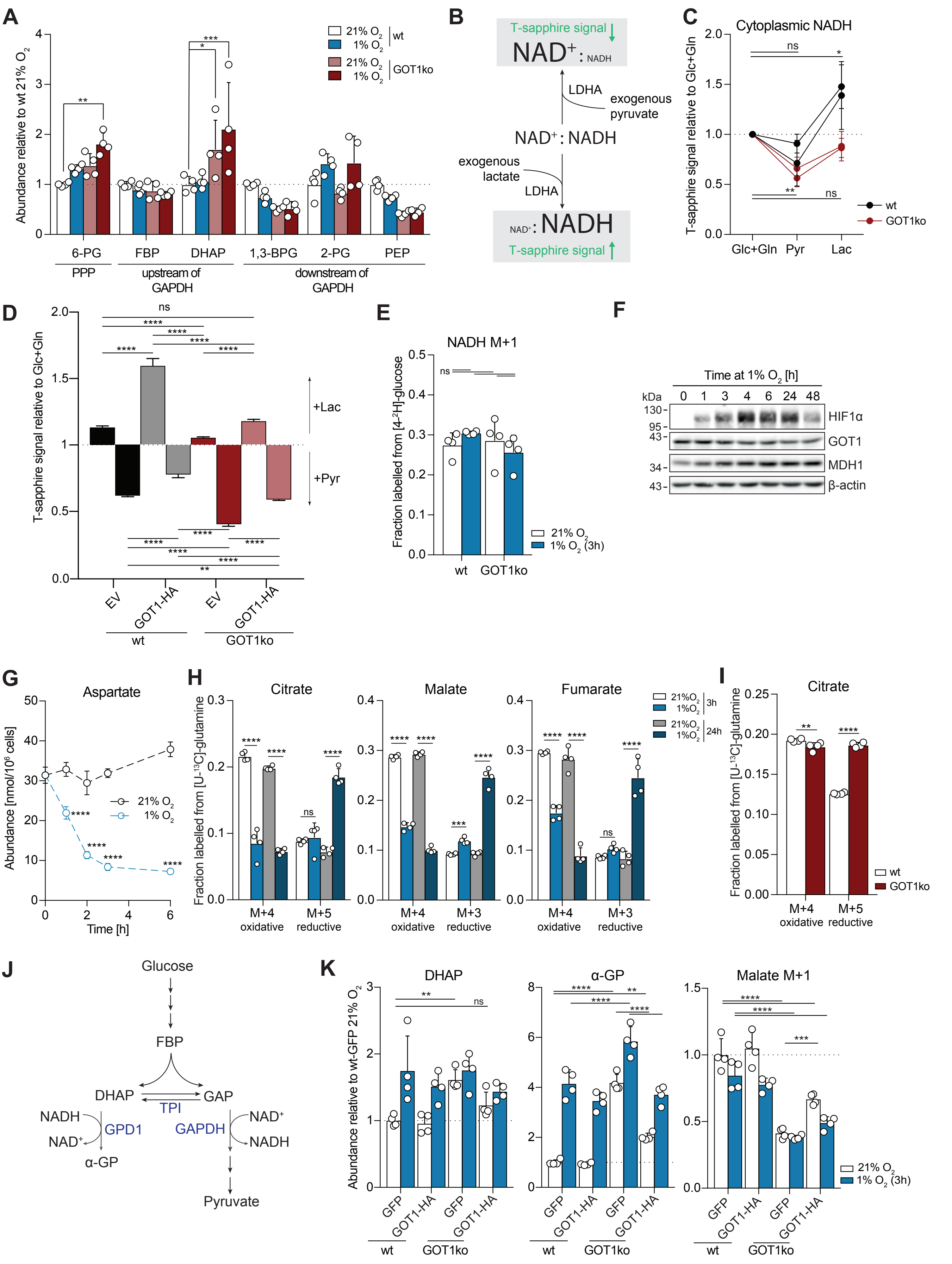

***Appendix Figure 4** - Figure legend on the next page*

Appendix Figure S4 related to Figure 4. Aspartate metabolism, and reductive carboxylation in early hypoxia.
A) Bar graph representation of the data in **Figure 4A**.
B) Schematic illustrating the effect of exogenous lactate and pyruvate on the cytoplasmic NAD^+/NADH ratio and Peredox T-sapphire fluorescence intensity.
C) Peredox T-sapphire fluorescence signal intensity in wild-type (wt) and GOT1ko MCF7 cells in buffer containing 5.5 mM glucose and 2 mM glutamine (Glc+Gln) and after sequential incubation first with 10 mM pyruvate (Pyr) and then with 10 mM lactate (Lac) (reverse order of that used in **Figure 4E**). Signal was normalized per nucleus and is shown relative to the Glc+Gln condition. See also **Figure 4E**.
D) Peredox T-sapphire fluorescence signal intensity in wild-type (wt) and GOT1ko MCF7 cells stably expressing an empty vector (EV) or HA-tagged GOT1. Cells in buffer containing 5.5 mM glucose and 2 mM glutamine (Glc+Gln) were sequentially incubated first with 10 mM lactate (Lac) and then with 10 mM pyruvate (Pyr). Signal was normalized per nucleus and is shown relative to the Glc+Gln condition.
E) Fraction of NADH labelled from [4-^{2}H]-glucose in the experiment shown in **Figure 4G**.
F) Western blot to assess levels of GOT1 and MDH1 in wild-type (wt) and GOT1ko cells incubated in 1% O_2 for the indicated lengths of time.
G) Intracellular abundance of aspartate in the experiment shown in **Figure 4H-J**.
H) Fractions of citrate, malate and fumarate labelled from [U-^{13}C]-glutamine after incubation with the tracer in 21% O_2 or 1% O_2 for 3 or 24 h. Isotopologues produced via oxidative TCA (citrate M+4, malate M+4, fumarate M+4) and via reductive carboxylation (citrate M+5, malate M+3, fumarate M+3) are shown.
I) Fraction of citrate labelled from [U-^{13}C]-glutamine after incubation of wild-type (wt) and GOT1ko MCF7 cells with the tracer for 3 h.
J) Schematic illustrating the fate of glucose carbons on either side of the triosephosphate isomerase (TPI) reaction.
K) Intracellular abundance of dihydroxyacetone phosphate (DHAP), α-glycerophosphate (α-GP) and malate M+1 (labelled from [4-^{2}H]-glucose after incubation with the tracer for 3 h) in wild-type (wt) and GOT1ko MCF7 cells stably expressing GOT1-HA or GFP after 3 h at 21% O_2 or 1% O_2.

Data information: Datapoints represent mean ± s.d. (A, C, E, G-I and K) or ± s.e.m. (D). n = 4 cultures for each time point/cell line/condition (A, C-E, G-I, K) except for G, 1% O_2, 2 h (n = 3) and 21% O_2, 3 h (n = 2). Data points in C represent mean ± SD of 2 independent replicates per cell line (n = 17-56 cells per replicate). Data points in D (n = 64-654 cells per cell line) are representative of 3 independent experiments. P values shown were calculated by two-way ANOVA Dunnett's test (C), two-way ANOVA Sidak's test [A, E, G-I, K (DHAP)], two-way ANOVA Tukey's test [K (α-GP, Malate M+1)], or Kruskal-Wallis test (D). ns: non-significant, * P<0.05, ** P<0.01, *** P<0.001, **** P<0.0001.

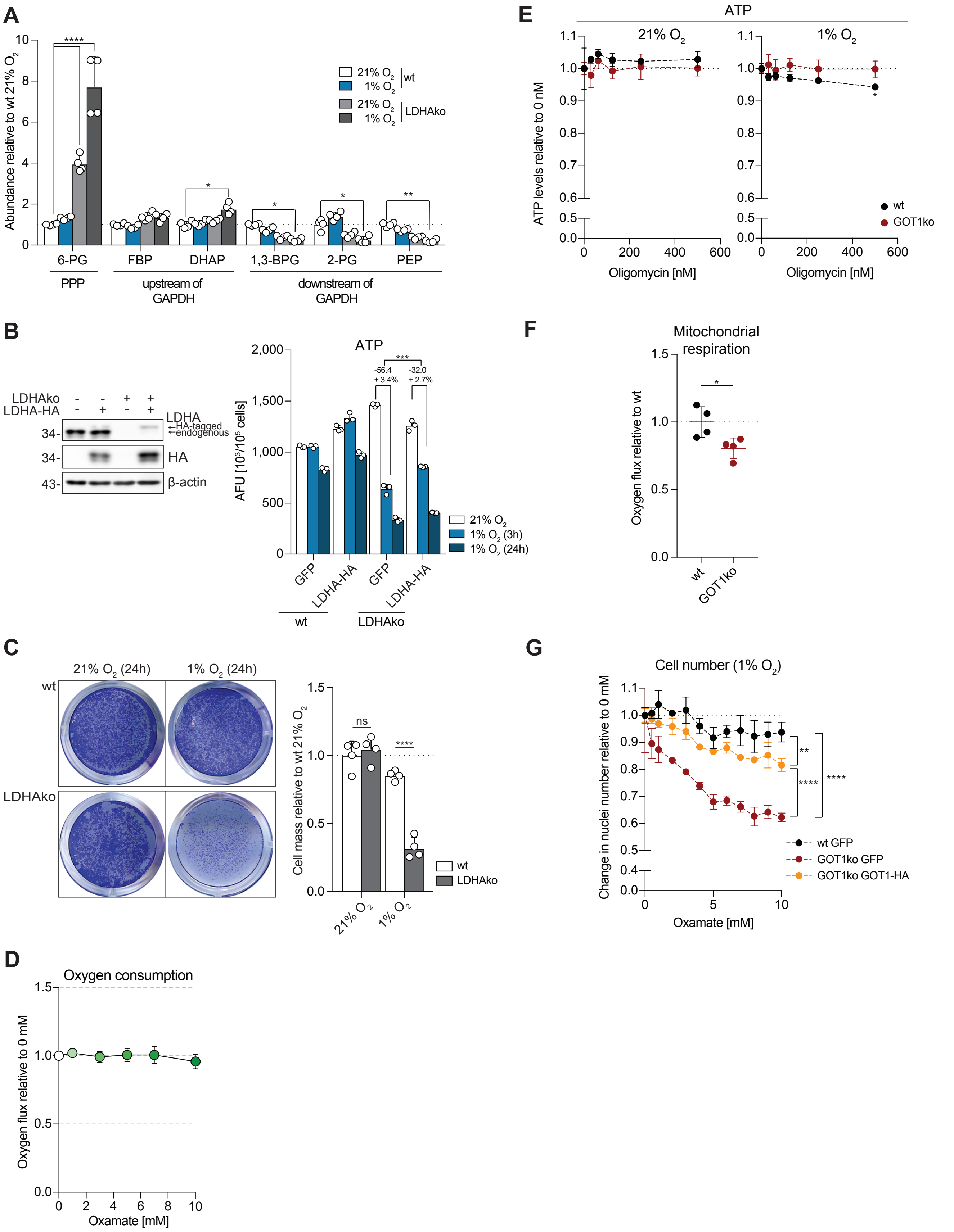

Appendix Figure 5 *- Figure legend on the next page*

Appendix Figure S5 related to Figure 5. Effects of LDHA knock-out on metabolism and cell mass accumulation.
A) Bar graph representation of the data in **Figure 5C**.
B) Left side: Western blot to assess the levels of endogenous LDHA and HA-tagged LDHA in wild-type and LDHAko MCF7 cells stably expressing LDHA-HA or GFP. Right side: ATP levels in the same cell lines at 21% O_2 and after 3 h or 24 h in 1% O_2. See also **Figure 5E**.
C) Cell mass accumulation in wild-type (wt) and LDHAko MCF7 cells assessed by crystal violet staining after 24 h at 21% O_2 or 1% O_2. Left side shows images of representative wells (with fixed and stained cells) from under each condition; right side shows quantification of crystal violet staining.
D) Cellular oxygen consumption in MCF7 cells treated successively with increasing oxamate concentrations.
E) ATP levels in wild-type (wt) and GOT1ko MCF7 cells at 21% O_2 and after 3 h in 1% O_2 treated with the indicated oligomycin concentrations for 3 h.
F) Mitochondrial respiration of wild-type (wt) and GOT1ko MCF7 cells. Cellular oxygen consumption was corrected for ROX (residual oxygen consumption) by addition of the complex III inhibitor antimycin A.
G) Change in cell confluence of wild-type (wt) and GOT1ko MCF7 cells stably expressing GOT1-HA or GFP within 24 hours at 1% O_2 with the indicated oxamate concentration in cell culture media, shown relative to 0 mM oxamate per cell line. Significance only shown for 10 mM [oxamate].

Data information: Datapoints in all panels represent mean ± s.d. n = 4 (A, F), n = 3 (C, E) cultures for each cell line and condition, or n = 3 (B) assays per cell line and condition. For D, graphs show combined replicate measurements from 2-3 independent experiments per condition. P values shown were calculated by two-way ANOVA Sidak's test (A), two-way ANOVA Tukey's test (C, G), one-way ANOVA Dunnett's test (D), two-way ANOVA Dunnett's test (E), or unpaired, two-tailed t-test (F). Statistical errors in B were propagated to calculate the error of the change in lactate between normoxia and hypoxia for each condition and significance between these changes was then tested using unpaired, two-tailed t-test. ns: non-significant, * $P<0.05$, ** $P<0.01$, *** $P<0.001$, **** $P<0.0001$.

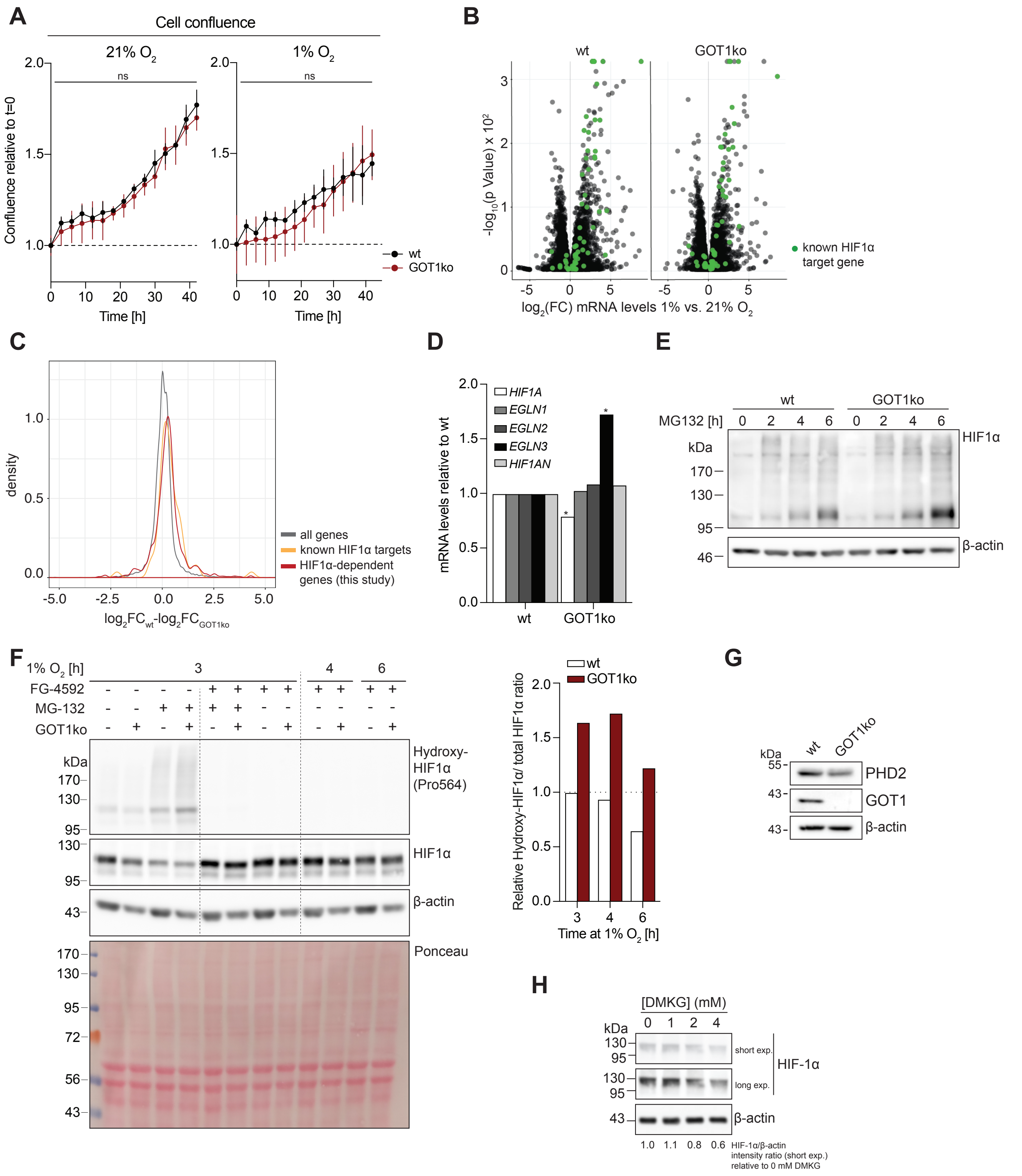

Appendix Figure 6 *- Figure legend on the next page*

Appendix Figure S6 related to Figure 6. HIF1α hydroxylation, stability and target gene expression in GOT1ko cells.

A) Cell confluence of wild-type (wt) and GOT1ko MCF7 cells at 21% O_2 or 1% O_2, shown relative to t=0 h. Time points also indicate duration of hypoxia treatment.

B) Volcano plot of gene expression changes in wild-type (wt) MCF7 and GOT1ko cells exposed to 1% O_2 for 24 h, compared to control cells in normoxia. HIF1α target genes shown in green.

C) Histogram showing frequency density of the difference in log_2-fold mRNA expression changes after 24 h in 1% O_2 between wild-type (wt) and GOT1ko MCF7. The graph shows the distribution of all genes, known HIF1α target genes and experimentally determined HIF1α-dependent genes from this study (See **Dataset EV1**). HIF1α-dependent genes are defined as protein-coding genes (excluding long non-coding RNAs, antisense RNAs and miRNAs), the expression of which changed more than 2-fold in wild-type MCF7 cells after 24 h at 1% O_2 but less than 2-fold in HIF1α^{mut} MCF7 cells.

D) Fold-change of expression of the indicated genes in GOT1ko cells relative to wild-type (wt) MCF7 cells from (B). *EGLN1*, *EGLN2*, *EGLN3* and *HIF1AN* encode PHD2, PHD1, PHD3 and factor inhibiting HIF (FIH), respectively.

E) Western blot to assess the levels of HIF1α in wild-type (wt) and GOT1ko MCF7 cells treated with the proteasome inhibitor MG-132 (10 µM) for the indicated lengths of time.

F) Western blot to assess the levels of HIF1α and hydroxylated HIF1α (Pro564) in wild-type (wt) and GOT1ko MCF7 cells exposed to 1% O_2 for the indicated lengths of time**.** Cells were treated with the PHD inhibitor FG-4592 (50 µM), the proteasome inhibitor MG-132 (10 µM) or a combination of both for the duration of the experiment. Right side shows quantification of lanes corresponding to MG-132–only treated samples from western blots in **Figure 6E** and **S6F**, presented as the ratio of hydroxylated HIF1α to total HIF1α. See also **Figure 6E**.

G) Western blot to assess the levels of PHD2 in wild-type (wt) and GOT1ko MCF7 cells.

H) Western blot to assess the levels of HIF1α in wild-type MCF7 cells incubated at 1% O_2 in media containing the indicated doses of the cell-permeable αKG analogue DMKG.

Data information: Datapoints in A represent mean ± s.d. n = 3 cultures for each cell line and condition (A, B). FDRs in B and derivative graph D were calculated using the ’exactTest’ function of the edgeR package (see Materials and Methods) with a cut-off set at 1%; only changes with FDR<0.01 are shown. Significance in A was calculated by two-way ANOVA Sidak’s test (ns: not significant). Asterisks in D indicate genes with FDR<0.01.

Appendix Table S1. Table of fragment ions used for metabolite quantification by GC-MS.

Metabolite	KEGG ID	m/z	Chemical Formula	Notes
3-Phosphoglycerate (3PG)	C00197	459		
Alanine	C00041	116		
Aspartate	C00049	334	C12 O4 N1 H28 Si3	
Citrate	C00158	375	C15 O5 H31 Si3	
Cysteine	C00097	220		
Fumarate	C00122	245	C9 O4 H17 Si2	
Glutamate	C00025	348	C13 O4 N1 H30 Si3	
α-Glycerophosphate (α-GP)	C03189	445	C14 O6 H38 Si4	
Glycine	C00037	174		
Isoleucine	C00407	158		
Lactate	C00186	117	C5 O1 H13 Si1	
Leucine	C00123	158		
Lysine	C00047	217		
Malate	C00149	245	C10 O3 H21 Si2	Used in ^{2}H-labelling experiments
Malate	C00149	335	C12 O5 H27 Si3	Used in ^{13}C-labelling experiments
Methionine	C00073	176		
myo-Inositol	C00137	318		
Ornithine	C00077	174		
Phenylalanine	C00079	218		
Proline	C00148	142		
Putrescine	C00134	174		
Pyruvate	C00022	174	C6 O3 N1 H12 Si1	
scyllo-Inositol, internal standard	C06153	318		
Serine	C00065	204		
Succinate	C00042	247	C9 O4 H19 Si2	
Threonine	C00188	320		
Tryptophan	C00078	218		
Tyrosine	C00082	218		
Uracil	C00106	241		
Valine	C00183	144		

Appendix Table S2. Table of fragment ions used for metabolite quantification by LC-MS.

Metabolite	KEGG ID	Polarity	m/z	Fragments	Chemical Formula
6-Phosphogluconate	C00345	-	275.0173	96.969	C6 O10 H13 P1
				78.959	
Fructose 1,6-bisphosphate	C00354	+	341.0033	96.970	C6 O12 H14 P2
				78.959	
Dihydroxyacetone phosphate	C00111	-	168.9908	96.970	C3 O6 H7 P1
				78.959	
1,3-Bisphosphoglycerate	C00236	-	264.9520	116.975	C3 O10 H8 P2
				78.959	
2-Phosphoglycerate	C00631	-	184.9857	78.958	C3 O7 H7 P1
				96.969	
				166.975	
Phosphoenolpyruvate	C00074	-	166.9751	78.958	C3 O6 H5 P1
