# Supplementary material for: Metabolic priming by multiple enzyme systems supports glycolysis, HIF1α stabilisation, and human cancer cell survival in early hypoxia
Source: EMBO J. 2024 Mar 14;43(8):1545–69. doi: 10.1038/s44318-024-00065-w (PMC11021510; doi:10.1038/s44318-024-00065-w)
Supplement: Supplementary file 7 — Source Data Fig. 5 [file 44318_2024_65_MOESM7_ESM.zip › Figure 5/5A/5A-blot.pdf]

Source Data for Figure 5A

Grimm *et al.*

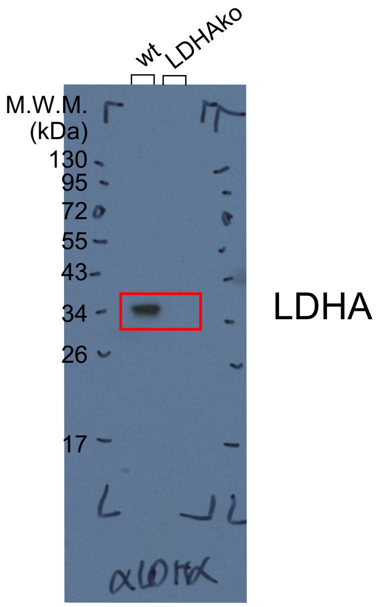

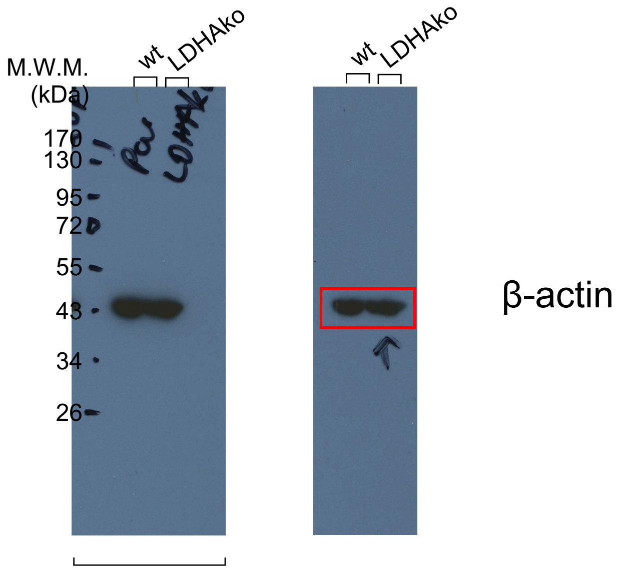

Different exposure, of the same membrane, than that used in the figure (on the right), showing position of molecular weight markers

M.W.M.: molecular weight markers

Red squares indicate cropped image parts used in the corresponding figure
