# Supplementary material for: Metabolic priming by multiple enzyme systems supports glycolysis, HIF1α stabilisation, and human cancer cell survival in early hypoxia
Source: EMBO J. 2024 Mar 14;43(8):1545–69. doi: 10.1038/s44318-024-00065-w (PMC11021510; doi:10.1038/s44318-024-00065-w)
Supplement: Supplementary file 8 — Source Data Fig. 6 [file 44318_2024_65_MOESM8_ESM.zip › Figure 6/6B/6B-blots.pdf]

Source Data for Figure 6B
Grimm *et al.*

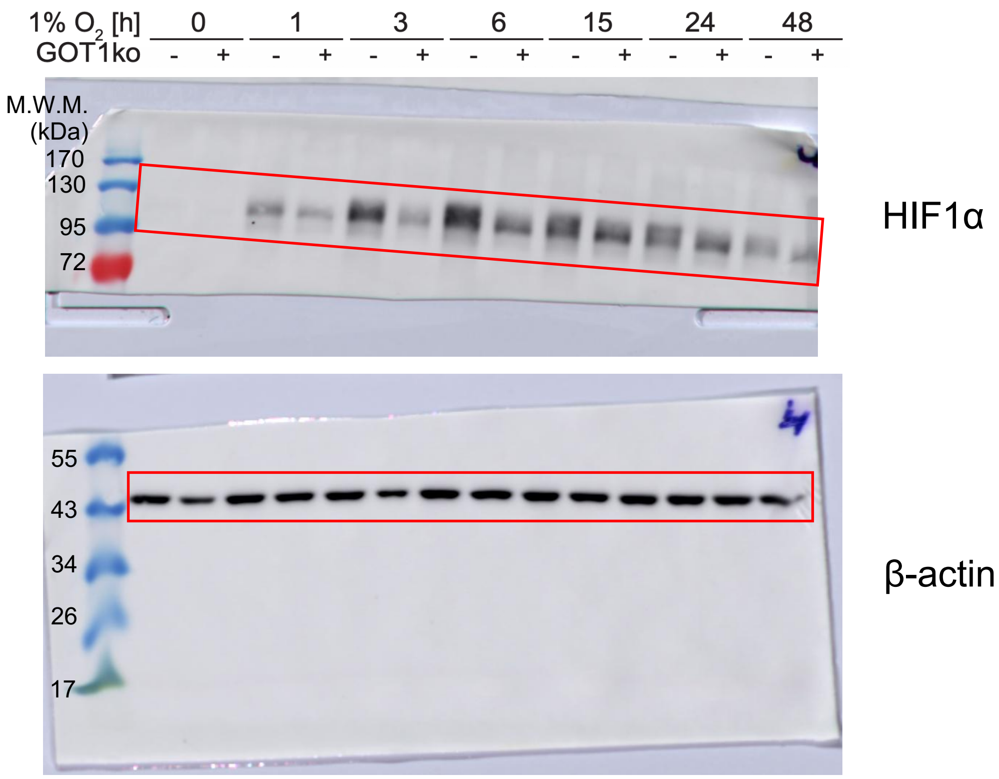

M.W.M.: molecular weight markers

Red squares indicate cropped image parts used in the corresponding figure
